# Addressing Commercial Health determinants: Indigenous Empowerment and Voices for Equity (ACHIEVE)—protocol for a multiphase study

**DOI:** 10.1136/bmjopen-2025-101735

**Published:** 2026-01-19

**Authors:** Beau Jayde Cubillo (Larrakia/Wadjigan), Jennifer Browne, Simone Sherriff, Troy Walker (Yorta Yorta), Karen Hill, Alessandro Crocetti, Fiona Mitchell, Kathryn Backholer, Raglan Maddox, Andrew Brown, Steven Allender, Cassandra J C Wright, Jennifer Lacy-Nichols, Catherine Chamberlain, Abe Ropitini (Ngāti Kahungunu/Ngāti Maniapoto iwi/Trawlwoolway), Nathan Taylor, Nadine Blair, Gregory Richards, Rachel R Huxley, Jaithri Ananthapavan, Joanne Hedges, Brianna Poirier, Anna Peeters, Phuong Nguyen, Boyd Swinburn, Sharon Atkinson-Briggs, Lee Yeomans, Yin Paradies

**Affiliations:** 1 Menzies School of Health Research, Darwin, Northern Territory, Australia; 2 Deakin University - Geelong Waterfront Campus, Geelong, Victoria, Australia; 3 The University of Sydney - Camperdown and Darlington Campus, Sydney, New South Wales, Australia; 4 Health and Social Development, Deakin University, Burwood, UK; 5 Australian National University, Canberra, Australian Capital Territory, Australia; 6 Global Obesity Centre, Deakin University School of Health and Social Development, Geelong, Victoria, Australia; 7 Faculty of Health, Deakin University, Melbourne, Victoria, Australia; 8 Centre for Alcohol Policy Research, La Trobe University, Melbourne, Victoria, Australia; 9 The University of Melbourne, Melbourne, Victoria, Australia; 10 Melbourne School of Population and Global Health, The University of Melbourne, Melbourne, Victoria, Australia; 11 Murdoch University, Murdoch, Western Australia, Australia; 12 VACCHO, Melbourne, Victoria, Australia; 13 Policy, Ethics, and Research, AH&MRC, Matraville, New South Wales, Australia; 14 NACCHO, Canberra, Australian Capital Territory, Australia; 15 Queensland Aboriginal and Islander Health Council, Brisbane, Queensland, Australia; 16 Faculty of Health, Deakin University, Burwood, Victoria, Australia; 17 The George Institute for Global Health, University of New South Wales, Sydney, New South Wales, Australia; 18 Economics, Cost-effectiveness, Deakin University, Burwood, Victoria, Australia; 19 Adelaide University, Adelaide, South Australia, Australia; 20 The University of Adelaide, Faculty of Health and Medical Sciences, Adelaide, South Australia, Australia; 21 Deakin University, Melbourne, Victoria, Australia; 22 Deakin Health Economics, Deakin University Faculty of Health, Burwood, Victoria, Australia; 23 University of Auckland - City Campus, Auckland, New Zealand; 24 Faculty of Arts and Education, Deakin University, Melbourne, Victoria, Australia

**Keywords:** health, Australian Aboriginal and Torres Strait Islander Peoples, qualitative research, health equity

## Abstract

**Abstract:**

**Introduction:**

The commercial determinants of health (CDoH) are a rapidly growing field of research and global health priority. Despite being disproportionately affected, Indigenous Peoples’ voices and perspectives are conspicuously absent from CDoH research and policy. This article outlines the protocol for Addressing Commercial Health determinants: Indigenous Empowerment and Voices for Equity (ACHIEVE), an Aboriginal and Torres Strait Islander-led project in Australia.

**Methods and analysis:**

ACHIEVE integrates four research streams, using a novel combination of methods. The first three streams will (i) conceptualise the CDoH using Indigenous yarning methodology, (ii) evaluate the effectiveness and cost-effectiveness of policies to reduce exposure to harmful marketing and (iii) assess the impacts of specific commercial entities on Aboriginal and Torres Strait Islander health using case studies. The final stream will consolidate findings from streams 1–3 and work with Aboriginal Community Controlled Health Organisations (ACCHOs) to co-create strategies for addressing the commercial determinants of Aboriginal and Torres Strait Islander health.

**Ethics and dissemination:**

Ethical approval for streams 1–3 has been granted by Deakin University Human Research Ethics Committee. ACHIEVE is guided by a governance model that prioritises Indigenous data sovereignty, community and ACCHO partnerships, capacity building and knowledge translation. Findings will be shared with participants, ACCHOs and policymakers to maximise research impact.

**STRENGTHS AND LIMITATIONS OF THIS STUDY**

- The project is Aboriginal-led, underpinned by robust theoretical frameworks and rigorous methods, combining Indigenous and western approaches in a culturally responsive manner.
- The Addressing Commercial Health determinants: Indigenous Empowerment and Voices for Equity (ACHIEVE) project is not designed to be representative of all Aboriginal and Torres Strait Islander Peoples and communities in Australia and the findings may not be generalisable.
- ACHIEVE upholds Indigenous data governance and sovereignty principles and focuses on collaboration between researchers and Aboriginal Community Controlled Health Organisations to co-create solutions to the commercial determinants of health.

## Introduction

 Although interactions with the private sector have long been considered within social models of health, the pathways through which commercial entities, products and practices influence health have been unified under the newer concept of ‘commercial determinants of health (CDoH)’.^1^ CDoH is now a rapidly growing field of research, as illustrated by recent new initiatives from WHO^2^ and the *Lancet*.^1^ Defined as ‘the systems, practices and pathways through which commercial actors drive health and equity’, it has been estimated that the CDoH are responsible for over 20 million deaths globally each year.^1^

Indigenous Peoples worldwide are often disproportionately affected by the products and practices of the commercial sector. For the purpose of this article, the term ‘Indigenous’ is respectfully applied to reference the global Indigenous Community and ‘Aboriginal and Torres Strait Islander’ is used to explicitly reference Indigenous Peoples of Australia. It is acknowledged these terms do not fully capture the unique linguistic, social, cultural and political diversity of Indigenous Peoples, and we respect the inherent rights and ongoing connections Indigenous Peoples have to the lands on which they reside.^3^

In Australia, commercial tobacco, unhealthy food and alcohol account for 20%, 15% and 12% of the gap in life expectancy between Aboriginal and Torres Strait Islander Peoples and other Australians, respectively,^4^ while the gambling and mining industries undermine social and cultural determinants of health and entrench inequity.^5 6^ While the commercial sector can produce significant harms, it also generates products, services and economic opportunities that are important for health and well-being. For example, the growing Aboriginal and Torres Strait Islander business sector is a major employer of Aboriginal and Torres Strait Islander Peoples and contributes to economic empowerment, community connection and self-determination. For this reason, we conceptualise commercial activity as having the potential to produce both benefits and harms. Although the commercial sector influences health in various ways, Aboriginal and Torres Strait Islander health policy remains largely focused on individual behaviours rather than commercial forces that drive outcomes.^7^ Aboriginal Community Controlled Health Organisations (ACCHOs) are beginning to lead health promotion initiatives that challenge this individual deficit focus.^8^,^10^ Recent research with ACCHOs in the state of Victoria revealed strong support for restricting marketing of harmful products, particularly gambling and ultra-processed foods, while advocating for greater investment in Aboriginal and Torres Strait Islander owned businesses.^6 10^

For many Indigenous Peoples, the intersecting forces of settler colonialism, capitalism, globalisation and neoliberalism have subjugated Indigenous Knowledges, values and lifeways, amplifying health disparities.^9 11 12^ Industries like tobacco, alcohol, gambling, mining, ultra-processed food and infant formula perpetuate colonial loss, eroding traditional practices and support networks.^5 11^ Yet, Indigenous Peoples have long resisted commercial exploitation. Examples include the campaign led by the Standing Rock Sioux Tribe against the construction of the Dakota Access Pipeline^13^ and, in Australia, the successful Aboriginal-led campaign against Woolworths’ proposed Dan Murphy’s alcohol megastore near ‘dry’ Aboriginal communities.^14^ Documenting and learning from these experiences offers critical insights for addressing commercial influences on Indigenous health and for enriching the CDoH field more broadly.

Despite the recent surge in CDoH research, Indigenous perspectives have been notably absent from this movement, limiting the field’s relevance to Aboriginal and Torres Strait Islander health policy and practice. It is therefore timely that the Australian National Health and Medical Research Council (NHMRC)^15^ invested $A7 million through a targeted call for research on the commercial determinants of Aboriginal and Torres Strait Islander health. This paper outlines the protocol for the Addressing Commercial Health determinants: Indigenous Empowerment and Voices for Equity (ACHIEVE) project, a 5-year research project.

### Aim and objectives

The ACHIEVE project aims to develop a novel, national, Aboriginal and Torres Strait Islander-led policy agenda to mitigate the negative health impacts and enhance the potential benefits of the commercial sector on Aboriginal and Torres Strait Islander health. To achieve this aim, the ACHIEVE project will:

1. Conceptualise Aboriginal and Torres Strait Islander perspectives on the CDoH and their interplay with social and cultural determinants of health.
2. Evaluate the effectiveness and cost-effectiveness of policies to mitigate the health impacts of harmful product marketing on Aboriginal and Torres Strait Islander Peoples.
3. Assess the health impacts of specific commercial entities on Aboriginal and Torres Strait Islander populations, including how the commercial sector influences policy development.
4. Work with Aboriginal and Torres Strait Islander leaders and the ACCHO sector to co-create strategies for mitigating the CDoH.

## Methods

### Research team

Centred on Aboriginal and Torres Strait Islander ways of knowing, being and doing, the ACHIEVE project was collectively conceptualised by an interdisciplinary team of Indigenous and non-Indigenous researchers, along with ACCHO representative organisations. ACHIEVE is led by senior Aboriginal (Wakaya) researcher (YP), with a team of 12 Indigenous investigators (YP, BJC, SS, TW, FM, RM, CC, AR, NT, SA-B, LY, JH), five representatives from the ACCHO sector (AR, NB, GR, LY, NT) and non-Indigenous investigators (JB, AC, KB, BP, AB, KH, SA, CJCW, JL-N, RRH, JA, AP, PN, BS, SA). The ACHIEVE team has expertise in a range of academic disciplines and experience working with the ACCHO sector. This includes expertise in Indigenous health and well-being (JB, SS, TW, BJC, RM, FM, BP, SA-B, NT), the CDoH (JB, JL-N, KB, SA, CJCW, AC) and the impact of racism, colonialism and neoliberalism on Indigenous health (YP and BP). Our investigators also bring specialised experience in researching the food (JB, KB, SS, BJC, TW, SA, JL-N, AP), alcohol (YP, CJCW, JB, AC, TW, JL-N, KB), tobacco, e-cigarette (vapes) and nicotine product (RM, SS, CC) industries, along with expertise in oral health (JH, BP), Indigenous methodologies (YP, SS, BJC, FM, TW, CC, RM), system science (SA, AB), public health policy analysis (JB, KB, BJC, SA, AP) and health economics (JA, PN). With investigators based in almost all states and territories of Australia, the team is well-equipped for a national-scale project.

### Patient and public involvement

The ACHIEVE project has been developed with Aboriginal and Torres Strait Islander leadership at its core, including partnerships with the ACCHO sector to ensure Community priorities are embedded into the research design, implementation and dissemination.^16 17^ Accordingly, the key partners for the ACHIEVE project are national, state and territory. Aboriginal organisations that represent the interests of the ACCHO sector within their respective jurisdictions include: The National Aboriginal Community Controlled Health Organisation (NACCHO), the Victorian Aboriginal Community Controlled Health Organisation, the Queensland Aboriginal and Islander Health Council, the Aboriginal Health and Medical Research Council of New South Wales (AH&MRC). Oversight of the research will be provided by a governance model that prioritises the needs and priorities of ACCHO partners, Indigenous data sovereignty, capacity building and knowledge translation.

### Study components

**Figure 1:**
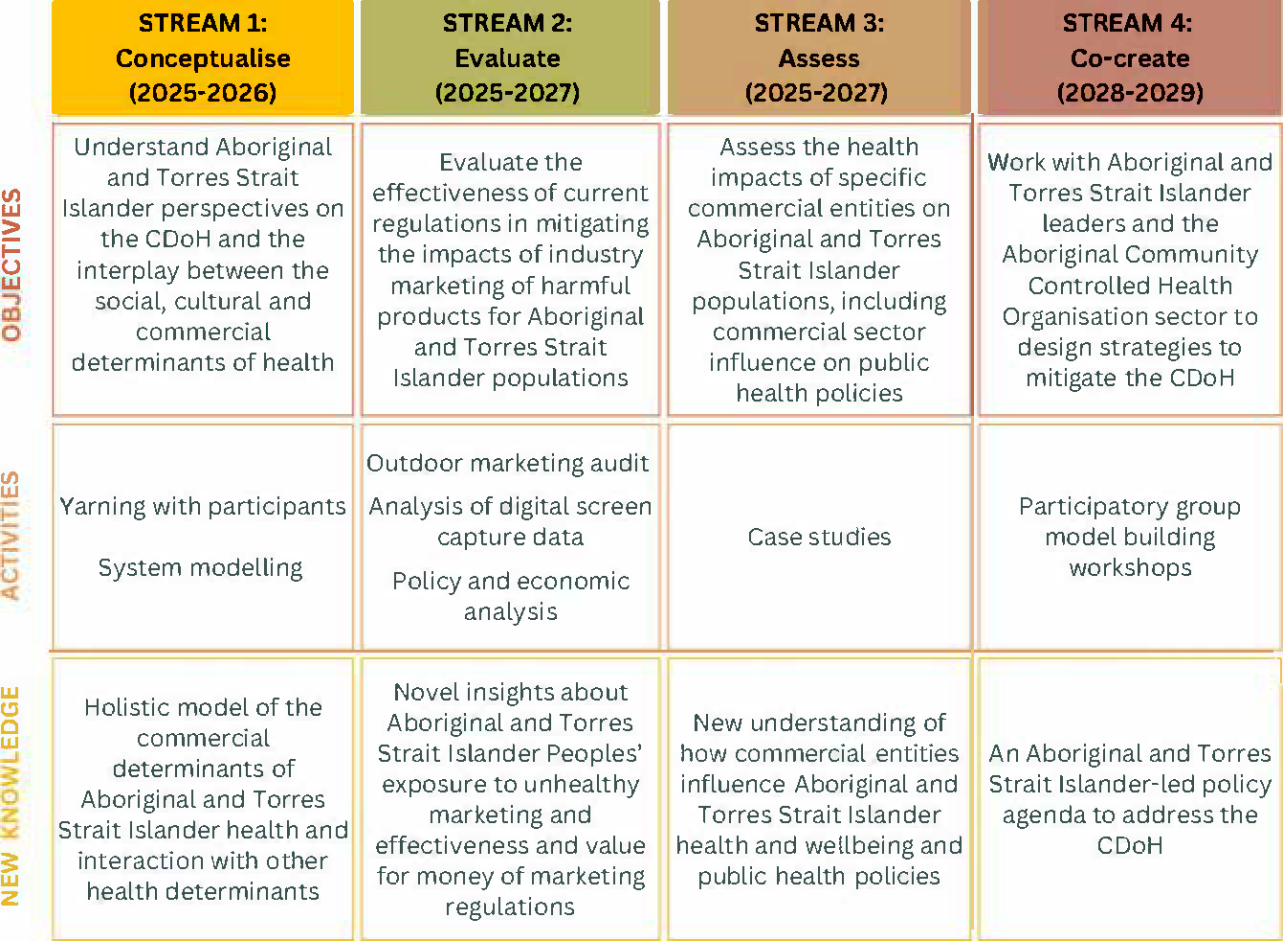
Addressing Commercial Health determinants: Indigenous Empowerment and Voices for Equity research streams. CDoH, commercial determinants of health.

ACHIEVE comprises four inter-related research streams aligned with the four project objectives (figure 1). The first three streams will be implemented concurrently during 2025–2027 to inform the co-creation of a CDoH policy agenda in 2028–2029 (stream 4).

### Stream 1: conceptualising the commercial determinants of Aboriginal and Torres Strait Islander health

#### Design

Stream 1 will engage with Aboriginal and Torres Strait Islander Peoples using yarning, a qualitative method theoretically underpinned by Indigenous ontology and epistemology.^18^ Yarning is a well-established, culturally safe and collaborative method that privileges Indigenous ways of knowing, being and doing and embodies principles of relationality, respect, flexibility and researcher accountability.^18 19^ Yarning is widely recommended for qualitative research with Aboriginal and Torres Strait Islander Peoples as it draws on cultural protocols and relationality to create a culturally safe space for participants to share stories from the position of their lived experience through Aboriginal communication styles.^8^

Yarning will be undertaken to elicit Aboriginal and Torres Strait Islander perspectives about the systems, practices and pathways through which commercial factors influence health and its determinants, either positively or negatively. Based on the advice of our partner organisations and Aboriginal team members in multiple states and territories, we will use purposive sampling to recruit at least 30 participants who can provide rich information about the CDoH. Snowball sampling will be used to recruit additional participants if needed. Our previous CDoH research in Victoria^6^ and the team’s extensive experience yarning with Aboriginal participants demonstrate that this is a feasible recruitment strategy. Participants will be Aboriginal and/or Torres Strait Islander adults, with the goal of recruiting a diverse range of Knowledge holders such as Community members from different geographical areas, Aboriginal and/or Torres Strait Islander staff from various sectors (eg, ACCHO, government, private sector, Aboriginal and/or Torres Strait Islander business).

### Data collection

A yarning topic guide has been co-developed by the research team and our partner organisations (online supplemental file 1). Yarns will be undertaken either in person or online (based on participant location and preference) and led by an Aboriginal member of the team, experienced in undertaking qualitative research with Aboriginal and Torres Strait Islander participants and familiar with the local context. If necessary, information about the study will be provided verbally and/or in local language. Each session will begin with informal social yarning to build trust, before moving to the research topic.^18^ While the focus of the yarns will be on understanding how commercial industries, products and practices affect Aboriginal and Torres Strait Islander health, how they unfold will largely be determined by the participants, with an emphasis on their priorities and interests.^8 18^ Consistent with our interest in understanding the full spectrum of CDoH, participants will be invited to discuss the ways commercial activities may both support and undermine health and well-being. With informed consent, yarning sessions will be audio-recorded and transcribed, and participants will be given the opportunity to review their transcript and change or add to their responses.

### Data analysis

Two forms of data analysis will be undertaken. First, de-identified transcripts will be uploaded into NVIVO software and coded inductively using reflexive thematic analysis.^20^ Following familiarisation with the transcripts, coding will be undertaken by Aboriginal researchers, who will pursue both surface-level and more nuanced meanings in the data. Codes will be grouped into thematic categories and then into interpretive themes through reflexive discussion with wider research team. Preliminary themes and interpretations will be further refined through collaborative yarning with ACCHO partners to ensure the findings reflect community perspectives.^18^

The second analysis strategy will employ principles of systems thinking,^21^ whereby transcripts will be coded for text that describes the causal linkages between variables (eg, commercial factors, social and cultural determinants, health risk factors and health outcomes). We will use this process to develop a system model using Deakin University’s purpose-built STICKE software.^22^ STICKE transforms coded variables and causal linkages into a visual system model that represents participants’ shared mental models of the interconnected factors within a complex system.^23^ International experts have called for system science tools to be used in the analysis of the CDoH,^24^ and our team has demonstrated the value and acceptability of systems thinking approaches in Aboriginal and Torres Strait Islander health research.^25^ This analysis, led by Aboriginal researchers with expertise in system modelling, will create the first holistic model of the commercial determinants of Aboriginal and Torres Strait Islander health and their interactions with other health determinants. The initial map will be refined via a workshop with ACCHO partners.

### Stream 2: evaluating the effectiveness of current marketing regulations

#### Design

Australians are increasingly exposed to marketing of harmful products, such as unhealthy foods and beverages, alcohol and gambling, across multiple media channels and personalised digital platforms. Marketing increases demand for commercial products and shapes social norms in ways that drive sales and consumption.^26^ Previous research identified unhealthy marketing, including online and within neighbourhood environments, as a key concern among Aboriginal and Torres Strait Islander communities.^6 10^ Yet the Australian regulatory landscape is dominated by voluntary codes and industry self-regulatory schemes, which have been widely criticised for their limited scope and weak accountability mechanisms.^27 28^ However, no research has examined the extent or nature of marketing exposure among Aboriginal and Torres Strait Islander Peoples, nor evaluated whether existing regulations are effective in reducing exposure to unhealthy product marketing.

In stream 2, we will quantify Aboriginal and Torres Strait Islander Peoples’ exposure to unhealthy outdoor advertising around schools and unhealthy online marketing. We will use these findings to determine whether existing codes and regulations for marketing commercial products are fit-for-purpose and model the likely effectiveness and cost-effectiveness for Aboriginal and Torres Strait Islander Peoples of policies that restrict unhealthy marketing.

### Outdoor marketing

We will assess the extent and nature of unhealthy food and alcohol outdoor advertisements within a 500 m radius of a sample of schools in 10 local government areas (LGA) with a high Aboriginal and Torres Strait Islander population and 10 LGAs with a low Aboriginal and Torres Strait Islander population. We will select two government schools from each LGA, matched on urban/regional location and on the socioeconomic index of the LGA as increased outdoor advertising has previously been observed in less affluent neighbourhoods.^29^ We will apply an existing protocol for monitoring outdoor marketing around schools.^30^ Advertisements will be categorised by size, type, location, content and product category. Differences in the total number and proportion of unhealthy advertisements (% of all advertisements) between schools in areas with high and low Aboriginal and Torres Strait Islander Peoples will be compared.

### Online marketing

We will quantify the nature and extent of Aboriginal and Torres Strait Islander Peoples’ exposure to online marketing of harmful products through an artificial intelligence (AI)-assisted analysis of screen capture data. We aim to recruit a convenience sample of approximately 100–200 Aboriginal and Torres Strait Islander adults, including at least 50 participants aged 18–25 years. This sample size reflects similar studies reporting on exposure to harmful online marketing.^31^ Participant recruitment will be conducted through the existing networks of the research team, our partner ACCHOs and via social media advertising. To be eligible, participants must: (1) live in Australia (we will begin recruitment in the state of Victoria and then extend nationally), (2) identify as Aboriginal and/or Torres Strait Islander, (3) be aged over 18 years, (4) use digital devices (eg, mobile phone, tablet, laptop) at least weekly, (5) have access to at least one digital device and social media account and (6) have sufficient English and digital literacy to read and understand the plain language statement and complete online research tasks. To determine whether eligibility criteria have been met and ensure informed consent, a member of the research team will meet participants who express interest in the study for an online introductory briefing, prior to the commencement of data collection.

### Data collection

Participants will be asked to screen-record their usual mobile device, ensuring recordings are captured on at least 1 weekday and 1 weekend day. We will ask participants to record for approximately 30% of the usual screen time each day (eg, if usual screen time is 2 hours, participants will record about 40 min) but no more than 1 hour. A recent study established that 30% of usual screen time is sufficient time to quantify exposure to unhealthy food advertising.^32^ Participants will be instructed to record their usual screen use (eg, browsing the internet and scrolling social media) but to stop recording during personal correspondence (eg, emails, chats, direct messages), financial transactions or any other private activity. Participants will have the opportunity to review, edit or delete their screen recordings before uploading them for analysis. Participants will also be asked to fill out a short (approximately 10 min) online survey to collect information about their usual screen time and online behaviour, consumption of unhealthy food and beverages, use of alcohol, tobacco/e-cigarettes and gambling and socio-demographic characteristics (online supplemental file 2).

### Data analysis

Members of our team have developed an AI-based image recognition system (SCANNER) that automatically identifies and extracts marketing images in video and photo data. Participants’ de-identified screen recordings will be processed using the SCANNER system to detect unhealthy marketing—this is an automated process that does not require researchers to view the recordings. SCANNER will analyse data to detect participants’ exposure to marketing of unhealthy food and beverages, tobacco, vapes, alcohol and gambling. Summary statistics will be reported to quantify participants’ exposure to different types of unhealthy marketing when online. Using survey data and descriptive statistics, we will calculate participants’ exposure to marketing from each specific sector using a validated method for measuring marketing exposure.^33^

### Policy and economic analysis

We will use findings to evaluate the effectiveness of current government regulations and industry codes of practice, which govern online and outdoor marketing in Australia. For all advertisements identified, we will examine whether they comply with relevant legislation and codes, including the Australian Association of National Advertisers Food and Beverage code,^34^ the Alcohol Beverages Advertising Code^35^ and Australia’s new Vaping Reforms Bill (2024).

Data on participants’ usual screen time and exposure to unhealthy marketing, both online and outdoors, will be used in an economic analysis to model the likely impact of policies designed to reduce exposure to marketing of unhealthy foods and beverages. Our team has adapted the validated ACE-Obesity Policy model^36^ to include demographic and epidemiological data and healthcare costs specific to the Aboriginal and Torres Strait Islander Peoples. We will use the exposure data generated in stream 2 and available literature to estimate the long-term health benefits of reducing exposure to unhealthy food and beverage marketing. This includes assessing the impact on the epidemiology of diet-related chronic disease (quantified as health-adjusted life years), healthcare cost savings and the incremental cost-effectiveness ratios. Our evaluation will compare the current regulatory environment with modelled policy scenarios for strengthening marketing regulations from a societal perspective.

### Stream 3: assessing the impacts of specific commercial entities on Aboriginal and Torres Strait Islander populations

#### Design

In stream 3, we will apply case study methodology^37^ to assess the health impacts of the key commercial industries or entities influencing Aboriginal and Torres Strait Islander health. Our team has previously undertaken CDoH case studies on Woolworths’ proposal to build an alcohol mega-store close to three ‘dry’ Aboriginal communities,^14^ and on the campaign to ‘free the Aboriginal flag’ from private ownership.^38^ Such case studies provide valuable insight into strategies employed by specific commercial entities as well as the power of Aboriginal and Torres Strait Islander-led advocacy and resistance. Our next case study will be on the influence of the pharmaceutical and pharmacy industries on Aboriginal and Torres Strait Islander health. We selected this topic as the pharmaceutical and pharmacy industry exerts significant influence on health policy, access to medicines and pharmacy service delivery in Australia. For Aboriginal and Torres Strait Islander Peoples, who often experience inequities in the healthcare system, the commercial power and practices of this industry have implications for health and well-being. Consultation with our ACCHO partners identified an interest in examining this sector from a CDoH perspective. During the course of the ACHIEVE project, further case studies may be undertaken on other industries based on the priorities of our partner organisations and the findings of the yarns undertaken in stream 1. All case studies will follow the methods outlined below.

### Data collection

Consistent with case study methodology,^37^ we will collect data from multiple sources, including government, non-government organisation (NGO) and industry documents, news articles and academic literature and key-informant interviews. We will search federal and state government and parliamentary websites to identify relevant policy documents, reports and submissions. Additionally, we will search these websites for records of ministerial diaries (available in four jurisdictions), lobbyist registers and political donation returns relevant to pharmaceutical or pharmacy industry. Previous CDoH research recommends these data sources for monitoring corporate political activity in Australia.^39 40^ We will search the websites of NGOs, including NACCHO, the Australian Medical Association and the Public Health Association of Australia for relevant policy statements and media releases.

To understand industry perspectives on both the potential beneficial and harms associated with the sector, we will review documents from leading pharmaceutical companies and pharmacy organisations. We will search for annual reports, policy statements, media releases on the Australian websites of the top 10 companies in these sectors, based on market capitalisation, as well as their industry representative organisations and professional associations (eg, Pharmacy Guild of Australia, Pharmaceutical Society of Australia, Medicines Australia). We will also search the Reconciliation Australia website to identify pharmaceutical and pharmacy industry Reconciliation Action Plans. Finally, we will search the Factiva, Medline and Google Scholar databases for media and academic articles related to the pharmaceutical or pharmacy industry and Aboriginal and Torres Strait Islander health. Search terms will be tailored to each website but will combine industry terms (eg, pharma*) with population terms (eg, Aboriginal* or “Torres Strait” or Indigenous).

To complement the documentary data, we will also undertake qualitative, semi-structured interviews to gather information from key informants to understand their perspectives on how the pharmaceutical and pharmacy industries impact Aboriginal and Torres Strait Islander health and well-being. Our purposive sampling strategy is designed to recruit participants who have diverse views, including perspectives on both the potential benefits and harms associated with these industries and their practices. Participants must be (1) over the age of 18 years, (2) live in Australia and (3) have experience in the area of Aboriginal and Torres Strait Islander health and/or the pharmacy/pharmaceutical sector as a policy maker, health practitioner, advocate, industry representative or as an Aboriginal or Torres Strait Islander community member/service user. We aim to recruit at least 10 participants from a range of jurisdictions and sectors who are likely to provide rich information about their experience with these industries. A semi-structured interview guide has been developed (see online supplemental file 3) based on our preliminary literature review and informed by frameworks for the CDoH.^1^ The guide includes questions exploring both positive and negative industry activities and their potential influence on Aboriginal and Torres Strait Islander health. Interviews will be conducted online by an Aboriginal member of the team and will be audio-recorded and transcribed.

### Data analysis

We have developed a data extraction template informed by frameworks on the CDoH^1 40^ and Corporate Health Impact Assessment (CHIA).^14^ CHIA is an established approach for analysing the potential health impacts of corporations to inform policy decision-making from an equity perspective.^41^ Interview data will be uploaded into NVIVO and coded using a reflexive thematic analysis approach.^42^ Coding will be inductive, with a focus on themes related to the systems, practices and pathways through which the pharmacy and pharmaceutical industry influences Aboriginal and Torres Strait Islander health. Preliminary themes and subthemes will be discussed among the research team until agreement is reached. These themes will be mapped against our adapted CHIA framework^43^ to assess the likely impacts of the industry on Aboriginal and Torres Strait Islander health and policy relevant to Aboriginal and Torres Strait Islander Peoples.

#### Stream 4: co-creating solutions to address commercial determinants of Aboriginal and Torres Strait Islander health

Throughout streams 1–3 of ACHIEVE, we will collectively identify key commercial entities and activities influencing Aboriginal and Torres Strait Islander health either positively or negatively, including the potential causal linkages between commercial determinants, social and cultural determinants and health outcomes. Concurrently, the research team and partners will collaboratively build a system dynamics model to synthesise the findings of the project’s initial three phases. System dynamics models provide a visual ‘map’ of the interconnected determinants of complex problems and provide insights on potential system changes.^23^ Our ACHIEVE system model will support the development of a policy agenda to address the CDoH.

In stream 4, we will hold participatory workshops in at least four states or territories with Aboriginal and Torres Strait Islander leaders, including representatives from the ACCHO sector. We will recruit up to 20 participants at each workshop through the research team’s existing networks, and through our ACCHO sector partners. The workshops will follow a systems thinking method called group model building,^23^ an established approach for working with communities to develop a shared understanding of the interrelated factors driving complex problems. Previous research from members of the ACHIEVE team has demonstrated that this method is a useful consensus building tool for co-creating community-led, system-level strategies to improve public health.^22^

Led by a trained Aboriginal facilitator, workshop participants will provide an overview of the key findings from streams 1–3. The system dynamics model will be presented so that participants can see how the key elements emerging from the research have been synthesised into a model of the commercial determinants of Aboriginal and Torres Strait Islander health. The presentation of the model will focus on visuals that depict the system structure, uncovered throughout the ACHIEVE project, and illustrate the hypothesised impacts of this system structure on Aboriginal and Torres Strait Islander health. Participants will refine the model until they are satisfied the diagram represents their shared understanding of the ways in which commercial activities influence health and well-being.

The second part of the facilitated workshop will identify policy and practice recommendations for countering the negative impacts and enhancing positive impacts of the commercial sector on Aboriginal and Torres Strait Islander health. Our STICKE software is specifically designed to build and edit system models in real time so participants can see how the recommendations emerging from the discussion will impact the system. Facilitators will guide participants through a prioritisation exercise to rank recommendations, based on potential impact and feasibility. Findings from the four jurisdictional workshops will be combined to produce Aboriginal and Torres Strait Islander-led system change agenda for ACCHOs and governments to address the commercial determinants of Indigenous health.

## Ethics and dissemination

Ethics approval for stream 1 has been granted by the Deakin University Human Research Ethics Committee (DUHREC) (#2025/HE000853) and the Aboriginal Health and Medical Research Council (AH&MRC) (#2377/25). Stream 2 has been approved by DHUREC (#2024/HE000219) and the Menzies School of Health Research (#2025/508). Stream 3 has been approved by DUHREC (#2024/HE000248) and AH&MRC (#2335/24). Separate ethics applications will be submitted to the relevant jurisdictional Aboriginal ethics committees, prior to the commencement of stream 4. All studies will be conducted in accordance with the National Health and Medical Research Council^44^ guidelines for ethical conduct in research with Aboriginal and Torres Strait Islander Peoples and communities and the Australian Institute of Aboriginal and Torres Strait Islander Studies Code of Ethics.^45^ Research protocols and data management plans have been approved by the ACHIEVE Indigenous Data Governance group. This committee will also approve research outputs prior to dissemination to uphold the principles of Indigenous data sovereignty. Guided by the ACHIEVE Knowledge Translation committee, findings will be shared with participants, ACCHOs and policymakers to maximise research impact. Academic outputs will adhere to the guidelines for ethical publishing in Indigenous contexts.^46^

## Discussion

The ACHIEVE project builds on and is informed by our team’s previous research in the CDoH for Aboriginal and Torres Strait Islander Peoples.^5 6 43^ This earlier work laid the foundation for establishing the research team, partners and methodology that ACHIEVE will now extend nationally. ACHIEVE will advance knowledge about Aboriginal and Torres Strait Islander health through Aboriginal and Torres Strait Islander-led research, generating new understandings of how commercial actors influence health. We will provide novel insights into Aboriginal and Torres Strait Islander Peoples’ exposure to unhealthy marketing, and the effectiveness and cost-effectiveness of regulations to reduce harmful marketing exposure. Additionally, we will undertake the first examination of how the pharmaceutical and pharmacy industry influences Aboriginal and Torres Strait Islander health and policy relevant to Aboriginal and Torres Strait Islander Peoples. ACHIEVE will produce the first holistic model of the commercial determinants of Aboriginal and Torres Strait Islander health and an Aboriginal and Torres Strait Islander-led policy agenda to address the CDoH.

Aboriginal and Torres Strait Islander leadership and governance is foundational to this work and will uphold Indigenous data sovereignty, maintain strong partnerships with the ACCHO sector and facilitate rapid, integrated knowledge translation. The ACHIEVE project will provide a strong foundation and support to further build Aboriginal and Torres Strait Islander research capacity, including through Aboriginal early career researchers who are leading all research streams. The project will strengthen the capacity of the ACCHO sector to address the CDoH in their health promotion strategies and advocacy. Such transformative research is essential to reducing commercial harms and improving Indigenous health outcomes, both in Australia and internationally.

## Supplementary material

10.1136/bmjopen-2025-101735 online supplemental file 1

10.1136/bmjopen-2025-101735 online supplemental file 2

10.1136/bmjopen-2025-101735 online supplemental file 3

## Data Availability

No data are available.

## References

[1] Gilmore AB, Fabbri A, Baum F (2023). Defining and conceptualising the commercial determinants of health. The Lancet.

[2] World Health Organisation (2023). Commercial determinants of health. https://www.who.int/news-room/fact-sheets/detail/commercial-determinants-of-health.

[3] Maddox R, Drummond A, Kennedy M (2024). Ethical publishing in ‘Indigenous’ contexts. Tob Control.

[4] Study AIoHaWABoD (2022). Australian burden of disease study: impact and causes of illness and death in aboriginal and torres strait islander people 2018.

[5] Crocetti AC, Cubillo Larrakia B, Lock Ngiyampaa M (2022). The commercial determinants of Indigenous health and well-being: a systematic scoping review. BMJ Glob Health.

[6] Crocetti AC, Walker T, Mitchell F (2024). Making Big Business Everybody’s Business: Aboriginal leaders’ perspectives on commercial activities influencing aboriginal health in Victoria, Australia. Global Health.

[7] Australian Government (2021). National Aboriginal and Torres Strait Islander Health Plan 2021-2031.

[8] Kennedy M, Maddox R, Booth K (2022). Decolonising qualitative research with respectful, reciprocal, and responsible research practice: a narrative review of the application of Yarning method in qualitative Aboriginal and Torres Strait Islander health research. Int J Equity Health.

[9] Poirier BF, Hedges J, Soares G (2022). Aboriginal Community Controlled Health Services: An Act of Resistance against Australia’s Neoliberal Ideologies. Int J Environ Res Public Health.

[10] Browne J, Walker (Yorta Yorta) T, Hill (Torres Strait Islander) K (2024). Food policies for Aboriginal and Torres Strait Islander health (FoodPATH): A systems thinking approach. Food Policy.

[11] Eisenkraft Klein D, Shawanda A (2024). Bridging the commercial determinants of Indigenous health and the legacies of colonization: A critical analysis. Glob Health Promot.

[12] Waa A, Robson B, Gifford H (2020). Foundation for a Smoke-Free World and healthy Indigenous futures: an oxymoron?. Tob Control.

[13] Cappelli ML (2018). Standing With Standing Rock: Affective Alignment and Artful Resistance at the *Native Nations Rise* March. Sage Open.

[14] Crocetti AC, Cubillo Larrakia B, Walker Yorta Yorta T (2023). A recipe for cultural disaster!’- a case study of Woolworths Group’s proposal to build an alcohol megastore in Darwin, Northern Territory. Global Health.

[15] Council NHaMR (2024). $7 million to target aboriginal and torres strait islander health. https://www.nhmrc.gov.au/about-us/news-centre/7-million-target-aboriginal-and-torres-strait-islander-health.

[16] Harfield SG, Davy C, McArthur A (2018). Characteristics of Indigenous primary health care service delivery models: a systematic scoping review. Global Health.

[17] Huria T, Palmer SC, Pitama S (2019). Consolidated criteria for strengthening reporting of health research involving indigenous peoples: the CONSIDER statement. BMC Med Res Methodol.

[18] Bessarab D, Ng’andu B (2010). Yarning About Yarning as a Legitimate Method in Indigenous Research. *IJCIS*.

[19] Atkinson P, Baird M, Adams K (2021). Are you really using Yarning research? Mapping Social and Family Yarning to strengthen Yarning research quality. *AlterNative: An International Journal of Indigenous Peoples*.

[20] Braun V, Clarke V, Hayfield N, Bager-Charleson S, McBeath A (2022). Supporting Research in Counselling and Psychotherapy: Qualitative, Quantitative, and Mixed Methods Research.

[21] Meadows D (2008). Thinking in Systems: International Bestseller: Chelsea Green Publishing.

[22] Hayward A, Sjoblom E, Sinclair S (2021). A New Era of Indigenous Research: Community-based Indigenous Research Ethics Protocols in Canada. J Empir Res Hum Res Ethics.

[23] Hovmand PS (2013). Group model building and community-based system dynamics process. Community Based System Dynamics: Springer.

[24] Knai C, Petticrew M, Mays N (2018). Systems Thinking as a Framework for Analyzing Commercial Determinants of Health. Milbank Q.

[25] Browne J, Walker T, Brown A (2021). Systems thinking for Aboriginal Health: Understanding the value and acceptability of group model building approaches. SSM Popul Health.

[26] Jones A, Lacy-Nichols J, Baker P (2021). Disrupting the commercial determinants of health.

[27] Reeve B (2016). Self-regulation of food advertising to children: an effective tool for improving the food marketing environment. Monash University Law Review.

[28] Jongenelis MI, Pierce H, Keric D (2021). Are Australian regulatory codes adequate in scope to protect youth from alcohol advertising?. Health Promot J Austr.

[29] Backholer K, Baum F, Finlay SM (2021). Australia in 2030: what is our path to health for all?. Medical Journal of Australia.

[30] Mackay S, Molloy J, Vandevijvere S (2017). INFORMAS protocol: outdoor advertising (school zones).

[31] Kelly B, Bosward R, Freeman B (2021). Australian Children’s Exposure to, and Engagement With, Web-Based Marketing of Food and Drink Brands: Cross-sectional Observational Study. J Med Internet Res.

[32] Nicholson E, Kelly B (2023). Establishing the Minimum Media Time Sample Required to Obtain Reliable Estimates of Children’s Digital Media Food Marketing Exposures. Curr Dev Nutr.

[33] Vaipuna T, Allison L, Bhasin A (2020). An objective methodology capturing online commodity marketing and other harms. Health Promot Int.

[34] AANA (2021). AAoNAFaBccc. https://aana.com.au/self-regulation/codes-guidelines/food-and-beverages-code/.

[35] Scheme TABAC (2023). The alcohol beverages advertising code scheme | the abac code. https://www.abac.org.au/about/thecode/.

[36] Ananthapavan J, Sacks G, Brown V (2020). Priority-setting for obesity prevention-The Assessing Cost-Effectiveness of obesity prevention policies in Australia (ACE-Obesity Policy) study. PLoS One.

[37] Yin RK (2014). Case study research: design and methods: thousand oaks.

[38] Hill K, Walker T, Mitchell F (2025). Pride not profit: a commercial determinants of indigenous health case study from australia.

[39] Lacy-Nichols J, Marten R, Crosbie E (2022). The public health playbook: ideas for challenging the corporate playbook. Lancet Glob Health.

[40] Lacy-Nichols J, Nandi S, Mialon M (2023). Conceptualising commercial entities in public health: beyond unhealthy commodities and transnational corporations. The Lancet.

[41] Anaf J, Baum F, Fisher M (2022). Assessing the health impacts of transnational corporations: a case study of Carlton and United Breweries in Australia. Global Health.

[42] Braun V, Clarke V (2006). Using thematic analysis in psychology. Qual Res Psychol.

[43] Crocetti AC, Cubillo Larrakia B, Hill Torres Strait Islander K (2023). Media coverage of commercial industry activities impacting Aboriginal and Torres Strait Islander health, 2018-2022. Health Promot Int.

[44] NHMRC (2018). Ethical Conduct in Research with Aboriginal and Torres Strait Islander Peoples and Communities.

[45] AIATSIS (2020). AIATSIS Code of Ethics for Aboriginal and Torres Strait Islander Research.

[46] Maddox R, Drummond A, Kennedy M (2024). Ethical Publishing in ‘Indigenous’ Contexts.

