# Supplementary material for: Addressing Commercial Health determinants: Indigenous Empowerment and Voices for Equity (ACHIEVE)—protocol for a multiphase study
Source: BMJ Open. 2026 Jan 19;16(1):e101735. doi: 10.1136/bmjopen-2025-101735 (PMC12820813; doi:10.1136/bmjopen-2025-101735)
Supplement: online supplemental file 1 [file bmjopen-16-1-s001.docx]

| Addressing Commercial Health-determinants Indigenous Empowerment and Voices for Equity (ACHIEVE)  Yarning guide |
| --- |
| Social Yarning  The yarning session will begin with social yarning which is not recorded. Social yarning includes introductions, social and cultural connections to establish relationality. It is not part of data collection. |
| Research Topic Yarning  The researcher will ask permission to begin recording. Below are examples of the types of questions and topics that are likely to guide the yarn. These are just examples. Researchers will be guided by participants on where they want to take the yarn. |
| Demographics  Before we start the yarn is it ok if I ask you a few questions about yourself?   1. Do you identify as Aboriginal, Torres Strait Islander or both? 2. What age bracket are you in e.g. 20s, 30s, 40s, 50s, 60s etc 3. What gender do you identify as? 4. What state or territory do you live? 5. Do you live in an urban, regional, rural or remote location? |
| 1. How about we start with a bit of a yarn about ourselves. [researcher to introduce themself] Could you share a little about yourself, maybe a little about what you do for work or in the community? |
| Today I’d like to yarn with you about the ways big corporations or private companies impact Aboriginal and Torres Strait Islander health and well-being. This includes physical, social, emotional and cultural wellbeing. Feel free to share whatever you want—there’s no right or wrong answers.  When we talk about ‘activities’ of corporations or private companies, we’re thinking about the things they do – whether its day-to-day stuff or even one-off actions that may affect our health, either positively or negatively. For example, this could be something companies do in Community, like sponsoring events, or bigger picture things like marketing that companies do to sell their products or make more profits. It’s all about how the commercial sector shapes our health and wellbeing. That’s what we mean by commercial determinants of health. Do you have any questions before we begin?   1. Can you share your thoughts on how the activities of corporations or private companies might affect health and wellbeing for our Mob? If you’ve got any examples, I’d love to hear them, including how this may have changed over time? 2. Let’s yarn about different industries. Are there certain industries you think have a big effect on our health? How so?"   Prompt: Examples could include industries like tobacco, alcohol, junk food, gambling or mining. But it could be other industries too, or companies that are doing positive things for health. Are there any that stand out to you? [Keep prompting “Anything else?” until all relevant industries discussed]   1. Which industries or companies do you think are most concerning when it comes to health for our Communities? Why? 2. Can you think of any industries or private companies that may be having a positive impact on l health and wellbeing for our Mob?   Prompt: Examples could include Aboriginal/Torres Strait Islander-owned businesses   1. Private companies can sometimes influence things over time like housing, healthcare, employment, prisons or our access to Land and Country. What are your thoughts on this?   Prompt:  - When things like housing, healthcare, prisons or even land are controlled by private companies, what kind of impact do you think it might have on our health and wellbeing?   1. Working for private companies can have its pluses and minuses. Let’s yarn about what it’s like for Mob in these workplaces- what’s good and what’s not so good?   Prompts:  - Have you ever worked in a private company? How did it affect your wellbeing?  - What about working for an Aboriginal/Torres Strait Islander business?  -Any ideas on how this may have been in the past compared to today?   1. Big companies sometimes try to influence government decisions and policies. What are your thoughts on how they do this and how it affects our health?   Prompts: Do you think private companies have a say in policies that impact our Communities? Can you give me some examples? Has this changed over time?   1. Reconciliation Action Plans (or RAPs) are something that many companies are doing these days. What are your thoughts on RAPs and how they might affect wellbeing for our Communities?   Prompts: How would you like to see the RAP process change in the future? Or What should companies be doing instead of RAPs to make a positive impact for our Mob?   1. After everything that we’ve yarned about today, what do you think needs to happen to reduce the negative impacts and boost the positive impacts that corporations or private companies have on our health or wellbeing? 2. What could be done to keep companies in check? 3. How could we better support Aboriginal/Torres Strait Islander businesses? 4. What role do you think Aboriginal Community Controlled Organisations can play in addressing the health impacts of big companies? |
| Is there anything else you would like to add before we finish up?  Is there anyone else that you think we should speak to? |
