# Supplementary material for: Addressing Commercial Health determinants: Indigenous Empowerment and Voices for Equity (ACHIEVE)—protocol for a multiphase study
Source: BMJ Open. 2026 Jan 19;16(1):e101735. doi: 10.1136/bmjopen-2025-101735 (PMC12820813; doi:10.1136/bmjopen-2025-101735)
Supplement: online supplemental file 2 [file bmjopen-16-1-s002.docx]

**#DigitalMob Post study online survey**

Thanks for participating in our study! Our team at Deakin University is doing research to learn about the ads you see online for things like junk food, alcohol, tobacco, vapes, and gambling. Your screen recordings will help us with this. Now that you have finished recording your screen, we have a few questions to ask you to understand a bit more about your screen time and online experiences.

**Instructions for participants:**

- Please complete the survey online. Most of the questions are multiple-choice.
- You're free to opt out of the survey if you prefer. Just click 'no' when asked if you'd like to participate.
- If you complete the survey, you will receive an additional $50 gift voucher to thank you for your time.

**Questions**

1. Do you agree to participate in the survey?
   1. Yes
   2. No
2. What is your postcode? – [text box]
3. What is your date of birth? - [text box]
4. What is your gender?
5. Female
6. Male
7. Non-binary
8. Other [enter free text]

**Section 01 – In this section we will ask you about your online and screentime activities that are NOT related to work or study**.

1. How long do you usually spend online, not including for work or study? (e.g., browsing the internet, using social media, gaming)?
   1. Up to 15 minutes
   2. Up to 30 minutes
   3. Up to 1 hour
   4. Up to 2 hours
   5. Up to 3 hours
   6. Up to 4 hours
   7. Up to 5 hours
   8. Up to 6 hours
   9. More than 6 hours
2. Please select the activities you do online and rank them from the one you spend the most time on to the one you spend the least time on.
   1. Scrolling social media (e.g. Facebook, Instagram, TikTok, SnapChat etc.)
   2. Playing online games (e.g. through apps, websites etc.)
   3. Watching YouTube videos
   4. Shopping online (e.g. for food, clothes or other items)
   5. Browsing the internet (general searching of the internet)
   6. Chatting with friends (e.g. through apps or websites)
   7. Other – please type in these activities.

If option (b) is selected,

How many **hours a day** do you usually spend playing online games [drop down list]?

1. Up to 1 hour
2. Up to 2 hours
3. Up to 3 hours
4. Up to 4 hours
5. Up to 5 hours
6. Up to 6 hours
7. More than 6 hours
8. On a typical **weekday**, how long do you spend watching **free-to-air** commercial TV (e.g. Channel 7, 9, 10, SBS) or **free-on-demand** commercial TV (e.g. 7plus, 9Now, 10Play, SBS On Demand)?
   1. None
   2. Up to 15 minutes
   3. Up to 30 minutes
   4. Up to 1 hour
   5. Up to 2 hours
   6. Up to 3 hours
   7. Up to 4 hours
   8. Up to 5 hours
   9. Up to 6 hours
   10. More than 6 hours
9. On a typical **weekend**, how long do you spend watching **free-to-air** commercial TV (e.g. Channel 7, 9, 10, SBS) or **free-on-demand** commercial TV (e.g. 7plus, 9Now, 10Play, SBS On Demand)?
   1. Up to 15 minutes
   2. Up to 30 minutes
   3. Up to 1 hour
   4. Up to 2 hours
   5. Up to 3 hours
   6. Up to 4 hours
   7. Up to 5 hours
   8. Up to 6 hours
   9. More than 6 hours

**Section 02 – In this section, we will be asking questions about your diet and lifestyle patterns.**

1. How often do you eat food from fast food restaurants (e.g., MacDonald’s, Hungry Jacks, Dominos, KFC, Subway)? [tick box]
   - - - Daily
       - Multiple times per week
       - Weekly
       - Multiple times a month
       - Monthly
       - Less than once a month
       - Never
2. How often do you eat packaged snacks such as potato chips, corn chips, Twisties, Cheezels, Burger Rings, popcorn, muesli bars, LCMs, etc? [tick box]
   - - - Daily
       - Multiple times per week
       - Weekly
       - Multiple times a month
       - Monthly
       - Less than once a month
       - Never
3. How often do you drink sweetened drinks such as soft drinks, energy drinks, sweetened or flavoured waters or sports drinks? [tick box]
   - - - Daily
       - Multiple times per week
       - Weekly
       - Multiple times a month
       - Monthly
       - Less than once a month
       - Never
4. How often do you have a drink containing alcohol?
5. Never
6. Monthly or less
7. 2–4 times a month
8. 2–3 times a week
9. 4 or more times a week

If answer is (a) never, skip to 10

If answer is any option from (b) to (e),

9.1. How many standard drinks containing alcohol do you have on a typical day?


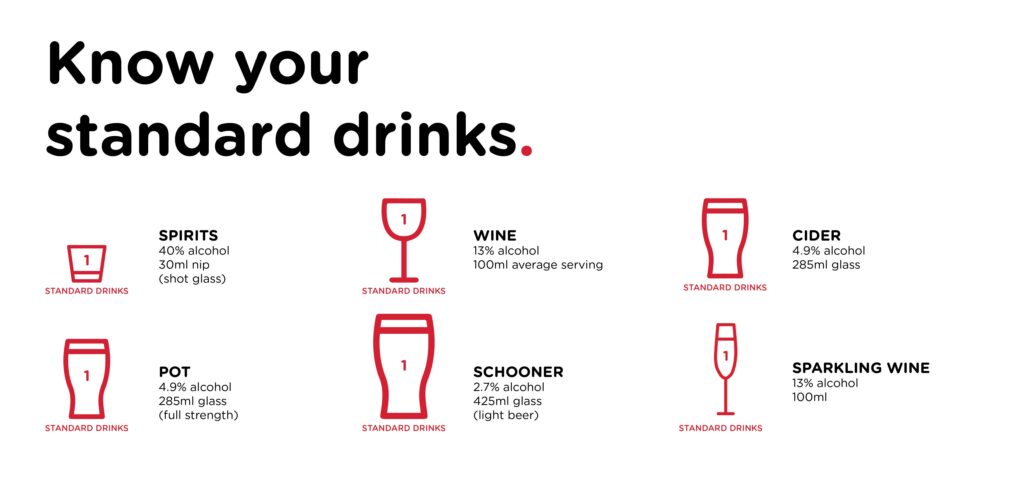


1. 1 or 2
2. 3 or 4
3. 5 or 6
4. 7 to 9
5. 10 or more

9.2. How often do you have six or more alcoholic drinks on one occasion?

1. Less than monthly
2. Monthly
3. Weekly
4. Daily or almost daily
5. How often do you bet on racing or sports matches, gamble at the casino, use poker machines, buy lottery tickets or gamble online?
   1. Never
   2. Less than 3 times a year
   3. Three times a year
   4. More than 3 times a year but less than monthly
   5. Once a month
   6. More than once a month
6. Do you smoke cigarettes or use e-cigarettes/vapes?
   1. No
   2. Yes I smoke cigarettes (or other tobacco products).
   3. Yes I use e-cigarettes/vapes.
   4. Yes I smoke and use e-cigarettes/vapes.
7. How many times have you used a vape?
8. Never
9. Just a few puffs
10. I have vaped less than 10 times in my life
11. I have vaped more than 10 times but less than 100 times in my life
12. I have vaped more than 100 times in my life

If c or d for Q12:

13a During the past 30 days, on how many days did use a vape or e-cigarette?

1. 0 days
2. 1-2 days
3. 3-5 days
4. 6-9 days
5. 10-19 days
6. 20-30 days

**Section 03 – The next set of questions are about things you buy online**

1. Do you consume foods ordered online or through a food delivery apps (e.g. UberEATS, Menulog, DoorDash etc)?
   1. Yes
   2. No

If yes,

14.1. Roughly how often? [tick box]

- - - - Daily
      - Multiple times per week
      - Weekly
      - Multiple times a month
      - Monthly
      - Less than once a month

14.2 Do you receive notifications or messages from these food delivery apps?

- 1. Yes
  2. No

If yes - roughly how many do you receive per week?

- 1. less than 1
  2. 1 – 2 times
  3. 3 – 4 times
  4. > 4 times

1. How often do you order junk food and/or soft drinks online?
   1. Often
   2. Sometimes
   3. Very rarely
   4. Never
2. How often do you order alcoholic drinks online?
   1. Often
   2. Sometimes
   3. Very rarely
   4. Never
3. How often do you gamble online or via apps?
   1. Often
   2. Sometimes
   3. Very rarely
   4. Never
4. How often do you purchase cigarettes or other tobacco products online?
   1. Often
   2. Sometimes
   3. Very rarely
   4. Never
5. How often do you purchase e-cigarettes and/or vapes online?
   1. Often
   2. Sometimes
   3. Very rarely
   4. Never

**Section 04 – This last section of questions is about your thoughts on online advertising of harmful products.**

1. Do you like seeing online advertising for following products:

| Junk food ads | Alcohol ads | Gambling ads | Cigarette or tobacco ads | Vape ads |
| --- | --- | --- | --- | --- |
| No, I don’t like seeing them and would like them to stop. | No, I don’t like seeing them and would like them to stop | No, I don’t like seeing them and would like them to stop | No, I don’t like seeing them and would like them to stop | No, I don’t like seeing them and would like them to stop |
| Yes, I don’t mind seeing them but I’d like to have the option to choose if I do or not. | Yes, I don’t mind seeing them but I’d like to have the option to choose if I do or not. | Yes, I don’t mind seeing them but I’d like to have the option to choose if I do or not. | Yes, I don’t mind seeing them but I’d like to have the option to choose if I do or not. | Yes, I don’t mind seeing them but I’d like to have the option to choose if I do or not. |
| Yes, I don’t mind seeing them. | Yes, I don’t mind seeing them. | Yes, I don’t mind seeing them. | Yes, I don’t mind seeing them. | Yes, I don’t mind seeing them. |
| Other – specify. | Other – specify. | Other – specify. | Other – specify. | Other – specify. |

1. Do you think government should restrict online advertising, especially for children and young people, for the following products?

| Junk food | Alcohol | Gambling | Cigarette and tobacco products | Vaping and e-cigarettes |
| --- | --- | --- | --- | --- |
| Yes | Yes | Yes | Yes | Yes |
| No – why | No – why | No – why | No – why | No -why |

- 1. Yes
  2. No – if no why?

1. Is there anything you like to share with us about your experience with online promotions or about this study?

This is the end of the survey. Thank you for your time in this study!
