# Supplementary material for: Addressing Commercial Health determinants: Indigenous Empowerment and Voices for Equity (ACHIEVE)—protocol for a multiphase study
Source: BMJ Open. 2026 Jan 19;16(1):e101735. doi: 10.1136/bmjopen-2025-101735 (PMC12820813; doi:10.1136/bmjopen-2025-101735)
Supplement: online supplemental file 3 [file bmjopen-16-1-s003.docx]

**Introduction**

Thank you for taking the time today to participate in our semi-structured interview. The interview will take approximately 60 minutes, it could run a bit longer or a bit shorter than this, is that okay with you?

This project is looking at the commercial industry and the ways they can affect the health – positively or negatively – of Aboriginal people across Australia. We are looking particularly at gaining a broader understanding of the impact and influence of the pharmacy and pharmaceutical industry on Aboriginal and Torres Strait Islander health.

We are especially interested to hear from people like yourself and others across multiple sectors and what kinds of experiences you have had, or are aware of, with the pharmacy and pharmaceutical industry and the ways in which they operate.

The pharmaceutical industry refers to the large multinational corporations that manufacture and distribute pharmaceutical products. Whereas the pharmacy industry refers to small and medium sized businesses that sell and distribute medications (often referred to as a pharmacy or a chemist in Australia).

Did you have any questions before we begin?

I just want to confirm with you that we are recording this interview to accurately portray the information you describe with us. I will start the recording now and will also be taking additional notes, are you happy for us to begin? Also, if you have any further questions or ideas that come to mind or you would prefer to change anything you mentioned we can be sure this is also honored, just let us know and we can organise for this to be changed or edited.

****Commence recording****

1. First can you tell us a little about yourself including your background and your past and present working experiences?
   1. Identify as Aboriginal or Torres Strait Islander? Sex/gender? Which sector(s) have you worked in (government, health, private industry, NFP, ACCHO, finance etc.)
2. What are your initial thoughts when you think of either the pharmacy pharmaceutical industry in Australia and the ways it impacts the health of Aboriginal and Torres Strait Islander people?
   1. Prompts: What are some of the positive things these industries do for Aboriginal health? What are some of the things these industries do that may negatively affect Aboriginal health? What feelings does the pharmaceutical industry conjure up for you here around health? Can you think of any specific examples of industry activity?
3. In your own experiences, what do you think the pharmacy or pharmaceutical industry are doing for Community members and the health of Aboriginal and Torres Strait Islander people?
   1. Prompts: What do you think industry might do well for Aboriginal health? Is there anything it doesn’t do well, or could it do better? In your opinion does the industry do anything harmful to Aboriginal health? Have you experienced or observed any negative intentions or actions from the industry?
4. Have you heard of some of the key pharmacy organisations in Australia, including the Pharmacy Guild of Australia (PGA) and the Pharmaceutical Society of Australia (PSA)?
   1. Prompts: If yes, ask about their knowledge, experiences with them and their intents and actions. If no, explain them briefly to participant and ask about their opinion on that thereafter.
5. You might be aware that it’s been recently announced by the federal government to increase the number of prescriptions dispensed at pharmacies across the country from 30 days to 60 days. What effect do you think this will have on the health and wellbeing of Aboriginal people?
   1. Prompts: In your opinion, is this something that will positively (or negatively) impact on you or your community?
6. Are you familiar with RAPs? And have you heard of some of the key pharmacy organisations in Australia including the Pharmacists Guild of Australia (PGA) and the Pharmaceutical Society of Australia (PSA)? If yes, what do you think of pharmaceutical companies, such as the Pharmacy having a Reconciliation Action Plan (RAP)?
   1. In your opinion, do RAPs make a difference?
   2. Do the progressions of the RAP (reflect, innovate, stretch and elevate) tell you there is progress with those pharmaceutical industries who have them?
7. We’d like to show you a short video from the PGA about Aboriginal health. [Show PGA video: <https://www.youtube.com/watch?v=6_N6USCfHOg>].
   1. What are your initial ideas about this video?
   2. What feelings and thoughts does this elicit from you?
8. Are you aware of or can you talk about any reported, known or professional experiences whereby there have been examples or stories of political donations and lobbying to governments by the pharmacy and pharmaceutical industry?
   1. Prompts: If yes, can you briefly explain what you experienced? What impact might you think this is having on health as it relates to Aboriginal people and a powerful commercial industry practice like pharmacy?
9. Are you aware of or can you talk about any current or recent pharmacy or pharmaceutical industry advertisements, marketing or media attention that comes to mind?
   1. Prompts: If yes, what do you recall about this? Did it target a particular audience (children, women, Aboriginal people, a certain illness or acute or chronic condition etc.)?
10. Did you have any final concluding thoughts about the pharmacy or pharmaceutical industry, or a take home message given your knowledge and experiences we can promote or discuss in future research work?
