# Supplementary material for: Addressing Commercial Health determinants: Indigenous Empowerment and Voices for Equity (ACHIEVE)—protocol for a multiphase study
Source: BMJ Open. 2026 Jan 19;16(1):e101735. doi: 10.1136/bmjopen-2025-101735 (PMC12820813; doi:10.1136/bmjopen-2025-101735)
Supplement: Supplementary file 4 [file bmjopen-2025-101735.reviewer_comments.pdf]

PEER REVIEW HISTORY

BMJ Open publishes all reviews undertaken for accepted manuscripts. Reviewers are asked to complete a checklist review form and are provided with free text boxes to elaborate on their assessment. These free text comments are reproduced below.

ARTICLE DETAILS

Title (Provisional)

Addressing Commercial Health-determinants- Indigenous Empowerment and Voices for Equity: Protocol for a multi-phase study

Authors

Cubillo (Larrakia/Wadjigan), Beau Jayde; Browne, Jennifer; Sherriff, Simone; Walker (Yorta Yorta), Troy; Hill, karen; Crocetti, Alessandro; Mitchell, Fiona; Backholer, Kathryn; Maddox, Raglan; Brown, Andrew; Allender, Steven; Wright, Cassandra J.C.; Lacy-Nichols, Jennifer; Chamberlain, Catherine; Ropitini (Ngāti Kahungunu/Ngāti Maniapoto iwi/Trawlwoolway, Abe; Taylor, Nathan; Blair, Nadine; Richards, Gregory; Huxley, Rachel R; Ananthapavan, Jaithri; Hedges, Joanne; Poirier, Brianna; Peeters, A; Nguyen, Phuong; Swinburn, Boyd; Atkinson-Briggs, Sharon; Yeomans, Lee; Paradies, Yin

---

VERSION 1 - REVIEW

**Reviewer**	**1**
**Name**	**Livingstone, Charles**
**Affiliation**	**Monash University**
**Date**	**09-Apr-2025**
**COI**	**None**

This is an important and timely project, and the protocol is well developed and likely to produce significant research data for exploration of the multiple issues that are associated with any inquiry into the Commercial Determinants of Health as they affect Indigenous people, in Australia and elsewhere. I have two minor comments. The description of 'Yarning' in the main body of the text might be expanded to better describe aspects of Indigenous information exchange. At present it is a little unclear how yarning might be preferred by Indigenous people as a means of providing detailed and relevant information. Secondly, I wonder whether consideration has been given to rendering the plain language statements and other project materials into Indigenous languages, to broaden the scope of data collection. For example, many Indigenous people from relatively remote communities visit population centres in, for example, the Northern Territory of Australia. When visiting such centres for medical, administrative or sporting purposes (all common reasons for travel)

some Indigenous people will be significantly affected by products that form part of the CDoH constellation (Gambling, alcohol, etc), and insights into their views may not be possible if English is the only language that people are presented with to enable participation in the study. Some Indigenous languages might be utilised if language speaking research staff are available. This may also mean attaining consent by verbal means. If this is feasible for the current team, I think it could enhance the validity of study data and findings if possible. Certainly, it could be readily adapted to the yarning method. Otherwise, I find the protocol compelling and very important, and its publication timely and significant.

Reviewer **2**

Name **Pulkki-Brännström, Anni-Maria**

Affiliation **Umeå University, Department of Public Health and Clinical Medicine, Epidemiology and Global Health**

Date **29-Sep-2025**

COI **None**

The protocol presents well an innovative, well-designed study. I have a few comments mainly relating to the motivation for the study and a few comments to clarify the methods.

My main comment relates to the inclusion of potential benefits of the commercial sector in the study. it is unclear what the motivation is for including potential benefits (alongside negative health impact) in the overall aim of the project. What those benefits might entail is vague because there are no references to previous studies about potential benefits. It seems benefits will be investigated within streams 1, 3 and 4 but not in 2, which could be clarified. In the appendices (yarning guide and interview guide), potential benefits are included, which could be mentioned more clearly in the main text. For the interviews in the case studies, if the sampling strategy is designed to be able to capture positive impacts, this should be explicitly stated. In summary, please clarify the motivation for including potential benefits in the study and how you plan to investigate those.

p7 in the Aim and objectives, “equity impact” is included in both streams 2 and 3. However elsewhere in the manuscript it is clear that equity impact is not included in stream 2. Thus the stated objective of stream 2 should be modified accordingly.

p7 in the second objective, “of” is missing

p8 and p17 As a reader unfamiliar with the Australian context, I was confused about the meaning of “peak” in “peak organisations”. I understand it now but please consider if you can add a short explanation to help international readers.

p9 The meaning of “2029-9” is not clear. Should it be 2028-2029?

Figure 1. The figure is very good. However on the row “Activities” sometimes you have just named the method, other times added a few words on how the method will be used. Compare “System modelling” and “Economic evaluation” with “Participatory workshops to identify and prioritise strategies”. As the figure has quite a lot of text, consider simplifying the row "Activities".

TImeline. Following journal guidelines, the dates of the study should be included in the manuscript. At the moment it is somewhat unclear what activities have already started.

p12 The rationale for and scope of stream 2 are not entirely clear. There is no clear statement of the knowledge gap that the analysis will address. Nor is it clear at all what characterises current Australian legislation and industry codes. (Some of this is hinted at in the appendix.) References to previous literature could help to better motivate this part of the study.

p15-16 In the policy and economic analysis, the comparators are not clear. Will you evaluate effectiveness and cost-effectiveness by comparing current regulations with inadequate compliance/implementation as (perhaps) observed in the empirical data, or with a hypothetical policy that is more “fit-for-purpose”?

p16 The motivation for choice of case study presented here, pharmaceutical and pharmacy industry, is rather weak: “Following consultation with ACHO partners…” A better motivation of its relevance for the study as a whole is warranted.

Reference nr 26: Please provide some more information to help find this publication, e.g. web address.

VERSION 1 - AUTHOR RESPONSE

**Reviewer 1**		
This is an important and timely project, and the protocol is well developed and likely to produce significant research data for exploration of the multiple issues that are associated with any inquiry into the Commercial Determinants of Health as they affect Indigenous people, in Australia and elsewhere. I have two minor comments. Otherwise, I find the protocol compelling and very	Thank you very much	N/A

important, and its publication timely and significant.		
The description of 'Yarning' in the main body of the text might be expanded to better describe aspects of Indigenous information exchange. At present it is a little unclear how yarning might be preferred by Indigenous people as a means of providing detailed and relevant information.	Thank you for this helpful suggestion. We have expanded our description of yarning to better explain why it is the preferred method for information exchange:<br>*Yarning is widely recommended for qualitative research with Aboriginal and Torres Strait Islander peoples as it draws on cultural protocols and relationality to create a culturally safe space for participants to share stories from the position of their lived experience through Aboriginal communication styles (Kennedy et al. 2022).*	Stream 1, para 1, p. 11
Secondly, I wonder whether consideration has been given to rendering the plain language statements and other project materials into Indigenous languages, to broaden the scope of data collection. For example, many Indigenous people from relatively remote communities visit population centres in, for example, the Northern Territory of Australia. When visiting such centres for medical, administrative or sporting purposes (all common reasons for travel) some Indigenous people will be significantly affected by products that form part of the CDoH constellation (Gambling, alcohol, etc), and insights into their views may not be possible if English is the only language that people are presented with to enable participation in the study. Some Indigenous languages might be utilised if language speaking research staff are available. This may also mean	Thank you for this suggestion. While it will not be feasible to translate plain language materials into every Indigenous language for this national study, we can certainly provide information verbally, in language if necessary, based on the advice of our local researchers undertaking yarning. We have made the following revision to the manuscript:<br><br>*Yarns will undertaken either in person or online (based on participant location and preference) and led by an Aboriginal member of the team, experienced in undertaking qualitative research with Aboriginal and Torres Strait Islander participants and familiar with the local context. If necessary, information about the study will be provided verbally and/or in local language.*	Stream 1, para 2. P.12

attaining consent by verbal means. If this is feasible for the current team, I think it could enhance the validity of study data and findings if possible. Certainly, it could be readily adapted to the yarning method.		
**Reviewer 2**		
The protocol presents well an innovative, well-designed study. I have a few comments mainly relating to the motivation for the study and a few comments to clarify the methods.	Thank you very much	N/A
My main comment relates to the inclusion of potential benefits of the commercial sector in the study. it is unclear what the motivation is for including potential benefits (alongside negative health impact) in the overall aim of the project. What those benefits might entail is vague because there are no references to previous studies about potential benefits. It seems benefits will be investigated within streams 1, 3 and 4 but not in 2, which could be clarified. In the appendices (yarning guide and interview guide), potential benefits are included, which could be mentioned more clearly in the main text. For the interviews in the case studies, if the sampling strategy is designed to be able to capture positive impacts, this should be explicitly stated. In summary, please clarify the motivation for including	Thank you for this constructive comment. We have now clarified the rationale for including potential positive impacts of the commercial sector in the project's aim and how we plan to investigate these.<br><br>We have added the following, including references about potential benefits, to the Introduction:<br><br>*While the commercial sector can produce significant harms, it also generates products, services and economic opportunities that are important for health and wellbeing. For example, the growing Aboriginal and Torres Strait Islander business sector is a major employer of Aboriginal and Torres Strait Islander peoples and contributes to economic empowerment, community connection and self-determination (Polidano et al, 2025; Eva et al, 2023). For this reason, we conceptualise commercial activity as having the potential to produce both benefits and harms.*<br><br>We have also clearly articulated how potential benefits will be explored in individual streams. For Stream 1, we have added the following under Data Collection:	Introduction Para 3, Page 5-6

potential benefits in the study and how you plan to investigate those.	*Consistent with our interest in understanding the full spectrum of CDoH, participants will be invited to discuss the ways commercial activities may both support and undermine health and wellbeing.*<br><br>In stream 2, the objective is explicitly about harmful product marketing so there are not benefits to be investigated.<br><br>In stream 3, we have made several additions to the data collection section clearly stating how the study design will capture benefits as well as harms:<br><br>*To understand industry perspectives on both the potential beneficial and harms associated with sector, we will review documents from leading pharmaceutical companies and pharmacy organisations.*<br><br>*Our purposive sampling strategy is designed to recruit participants who have diverse views, including perspectives on both the potential benefits and harms associated with these industries and their practices.*<br><br>*The guide includes questions exploring both positive and negative industry activities and their potential influence on Aboriginal and Torres Strait Islander health.*<br><br>In Stream 4 we have emphasised:<br><br>*ACHIEVE will collectively identify key commercial entities and activities influencing Aboriginal and Torres Strait Islander health either positively or negatively, including the potential causal linkages between commercial determinants, social and cultural determinants and health outcomes*	Stream 1, data collection, page 12<br><br>Stream 3, data collection, para 2, p. 18<br><br>Stream 3. Data collection, para 3, p. 18<br><br>Stream 3. Data collection, para 4, p. 19<br><br>Stream 4, para 1, p. 20

p7 in the Aim and objectives, "equity impact" is included in both streams 2 and 3. However elsewhere in the manuscript it is clear that equity impact is not included in stream 2. Thus the stated objective of stream 2 should be modified accordingly.	We agree with the reviewer's assessment and have removed "equity impact" from both objectives 2 and 3, as well as in the Stream 2 and 3 text.	Aim and objectives, p. 7
p7 in the second objective, "of" is missing	Thank you. We have added the word "of"	Aim and objectives, p. 7
p8 and p17 As a reader unfamiliar with the Australian context, I was confused about the meaning of "peak" in "peak organisations". I understand it now but please consider if you can add a short explanation to help international readers.	Thank you for pointing this out. We have provided this explanation for Aboriginal peak organisations:<br><br>*national, state and territory Aboriginal organisations who represent the interests of the ACCHO sector within their respective jurisdiction*<br><br>We have replaced the term "peak" with *"ACCHO representative organisations"*<br>And<br>*"industry representative organisations and professional associations*"	Patient and public involvement p. 9<br><br>p. 8<br><br>p. 18
p9 The meaning of "2029-9" is not clear. Should it be 2028-2029?	Thank you, this has been changed to 2028-2029	
Figure 1. The figure is very good. However on the row "Activities" sometimes you have just named the method, other times added a few words on how the method will be used. Compare "System modelling" and "Economic evaluation" with "Participatory workshops to identify and prioritise strategies". As the figure has quite a lot of text, consider simplifying the row "Activities".	Thank you for this suggestion we have updated Figure 1 to simplify the Activities row by simply naming the research methods.	Figure 1

TImeline. Following journal guidelines, the dates of the study should be included in the manuscript. At the moment it is somewhat unclear what activities have already started.	Thank you for this suggestion we have updated Figure 1 so that timelines are now included for each stream	Figure 1
p12 The rationale for and scope of stream 2 are not entirely clear. There is no clear statement of the knowledge gap that the analysis will address. Nor is it clear at all what characterises current Australian legislation and industry codes. (Some of this is hinted at in the appendix.) References to previous literature could help to better motivate this part of the study.	We have added the following paragraph at the beginning of Stream 2 to provide a clear rationale, including characterisation of current Australian regulatory codes, and statement of the knowledge gap that this stream seeks to address:<br><br>*Australians are increasingly exposed to marketing of harmful products, such as unhealthy foods and beverages, alcohol and gambling, across multiple media channels and personalised digital platforms. Marketing increases demand for commercial products and shapes social norms in ways that drive sales and consumption (Jones et al., 2021). Previous research identified unhealthy marketing, including online and within neighbourhood environments, as a key concern among Aboriginal and Torres Strait Islander communities (Browne et al. 2024; Crocetti et al. 2024). Yet the Australian regulatory landscape is dominated by voluntary codes and industry self-regulatory schemes which have been widely criticised for their limited scope and weak accountability mechanisms (Reeve 2016; Jongenelis et al., 2021). However, no research has examined the extent or nature of marketing exposure among Aboriginal and Torres Strait Islander peoples, nor evaluated whether existing regulations are effective in reducing exposure to unhealthy product marketing.*	Stream 2, para 1, p.13
p15-16 In the policy and economic analysis, the comparators are not clear. Will you evaluate effectiveness and cost-	We revised this section to make the comparison clearer. We have added the following text:	Policy and economic

effectiveness by comparing current regulations with inadequate compliance/implementation as (perhaps) observed in the empirical data, or with a hypothetical policy that is more "fit-for-purpose"?	*...model the likely impact of policies designed to reduce exposure to marketing of unhealthy foods and beverages*<br>and:<br>*Our evaluation will compare the current regulatory environment with modelled policy scenarios for strengthening marketing regulations*	analysis, para 2, p. 17
p16 The motivation for choice of case study presented here, pharmaceutical and pharmacy industry, is rather weak: "Following consultation with ACHO partners..." A better motivation of its relevance for the study as a whole is warranted.	We have added further rationale for selection of the pharmacy and pharmaceutical industry for examination in Stream 3:<br>*We selected this topic as pharmaceutical and pharmacy industry exerts significant influence on health policy, access to medicines, and pharmacy service delivery in Australia. For Aboriginal and Torres Strait Islander peoples, who often experience inequities in the healthcare system, the commercial power and practices of this industry have implications for health and wellbeing. Consultation with our ACCHO partners identified an interest in examining this sector from a CDoH perspective.*	Stream 3, para 1, page 18
Reference nr 26: Please provide some more information to help find this publication, e.g. web address.	We have added a doi link for this reference	Reference list
